# Challenges in designing interventions for food insecure families with food allergy in a Californian latinx cohort

**DOI:** 10.3389/falgy.2024.1389687

**Published:** 2025-01-16

**Authors:** Marleni Albarran, Emily Brown, Erin Martinez, Andrew R. Chin, Sayantani B. Sindher, Christopher M. Warren, R. Sharon Chinthrajah

**Affiliations:** ^1^ Department of Medicine, Sean N. Parker Center for Allergy and Asthma Research at Stanford University, Stanford, CA, United States; ^2^ Attane Health, Kansas City, MO, United States; ^3^ Food Equality Initiative, Lenexa, KS, United States; ^4^ Department of Preventive Medicine, Center for Food Allergy & Asthma Research, Northwestern University Feinberg School of Medicine, Chicago, IL, United States

**Keywords:** food allergy, food insecurity, research intervention, study design, underserved communities

## Abstract

Food allergy poses substantial social, economic, and quality of life burdens which are even heavier for families that are struggling with food insecurity. In the United States (US), food insecurity disproportionately affects vulnerable and historically marginalized communities, such as Latino/a/x and Black households. Targeting these disparities via our recent Food Equality Initiative (FEI) research intervention was challenging due to the barriers faced by the target underserved populations, which included poor digital literacy, language barriers, and limited access to necessary resources. These barriers hindered our efforts to promote access to nutritious and safe food options for food-insecure families, potentially further exacerbating health disparities. Here we discuss common challenges and opportunities associated with conducting research interventions in underserved communities in the US—leveraging our experiences designing and implementing an intervention to improve food allergy management through supplemental nutrition assistance in a predominantly Spanish-speaking, lower-income neighborhood in Northern California. We also provide recommendations for other researchers regarding how to tailor research strategies to address these challenges, and in so doing reduce health disparities and promote positive health outcomes for vulnerable and historically marginalized communities.

## Introduction

Food allergy (FA) is often a life-long disease, with the standard of care being allergen avoidance. Because many of the top allergens such as milk, egg, peanuts and tree nuts, wheat, and soy are found in common foods, food allergic families have limited food options and eating outside carries the risk of allergic reactions due to the potential for the unexpected inclusion of allergens (1, 2). Additionally, among children with FA, 43%–86% are allergic to more than 1 food (3–6), resulting in a food cost-increase of 5.8%–16.7% per allergen (7). On average, there is a food-cost increase of 9.8% for families, 18% for couples without children and 36.0% for a single-person household on a 6-food avoidance diet compared to a standard diet. In the US, the total societal cost of pediatric FA alone was estimated to be nearly US $25 billion in 2013 (8), with an even greater economic impact imposed over the past decade. These heavy financial burdens have notable racial disparities (9) and are especially challenging for households experiencing food insecurity (FI), which are households that report unreliable access to adequate, affordable, and nutritious food options (10).

About 23% of US households experience FI with wide racial disparities (11, 12). Latino/a/x households and Black households are roughly 2–3 times more likely to be affected by FI in comparison to White and Asian households (13, 14). During the COVID-19 pandemic, increased unemployment, poverty rates and decreased access to regular school meals due to COVID safety guidelines, exacerbated FI, particularly amongst the Hispanic/Latinx community, increasing rates from 7.8% in 2019 to 12.2% in 2020 (15–17). While some states in the US provided school meals for all school aged children and families during the pandemic, many state and federal food assistance programs do not fully accommodate individuals with many common food allergies.

Food assistance programs like Women, Infants and Children (WIC) offer some support to FI families, but WIC's current substitution framework may have limited benefit for FI families with FA (18). For example, while soy milk is a cow milk substitution, this does not support children with soy allergies. Currently, WIC does not offer many alternatives for those with egg, vegan, or gluten-free diets. While programs like WIC are a great resource for families facing FI, there is an urgent need to improve the availability of resources for FI families with FA.

We recently performed a research intervention aimed to support FI and FA mother-child dyads by leveraging Attane Health's (AH) diverse stock of allergen-safe food options that can be delivered free of charge directly to participant's homes. In collaboration with a local federally qualified health center (FQHC), we enrolled 38 families with food-allergic children to engage in this 6-month intervention. In terms of the recruitment process, a clinician at the FQHC utilized the electronic health record to identify possible FI families. Then, a clinical research coordinator performed a phone call introducing the study and if the family was interested, performing a FI screen thereafter. All participants had at least one physician-confirmed food allergy diagnosis, with about half diagnosed by their pediatrician, and a third allergist-diagnosed. Shellfish, peanut, and egg allergies were most common, but patients with finned fish, tree nut, milk, and wheat allergies were also represented. Roughly 2/3 of participants had at least one comorbid atopic conditions.

Each participant received a $200 monthly stipend in the form of FFM credit to self-select their unique allergen-safe groceries. The FEI intervention also provided $40-worth of fresh produce baskets from a local community supported agriculture (CSA) organization that supplemented the participant's diet. In addition to these allergen-safe food options, the FEI project captured study outcomes through baseline, 3-, 6-, and 9-month surveys. These surveys inquired about the patient's FA and medical history, basic household information, global stress, knowledge regarding FA, FA-related quality of life, epinephrine carriage practices, health care utilization, FA management self-efficacy, economic impact of FA, and the family's food security status. Outcome data from these surveys will be reported in a forthcoming manuscript. This study cohort mostly consisted of Hispanic/Latinx families who were predominantly Spanish-speaking (74%), residing in a low-income community. Of the 38 families who were initially enrolled, 20 families completed 6 consecutive months of FFM orders, while 21 completed 3 months of FFM orders and 32 families placed at least one order. Of these families, 27 completed the baseline intake questionnaire, whereas 17 completed the 3-month follow-up questionnaire and 19 completed the 6-month follow-up questionnaire. Retaining and providing ongoing support to these underserved communities came with additional challenges that were not always readily apparent, especially in the context of research.

## Barriers in targeting an underserved community

**Figure 1:**
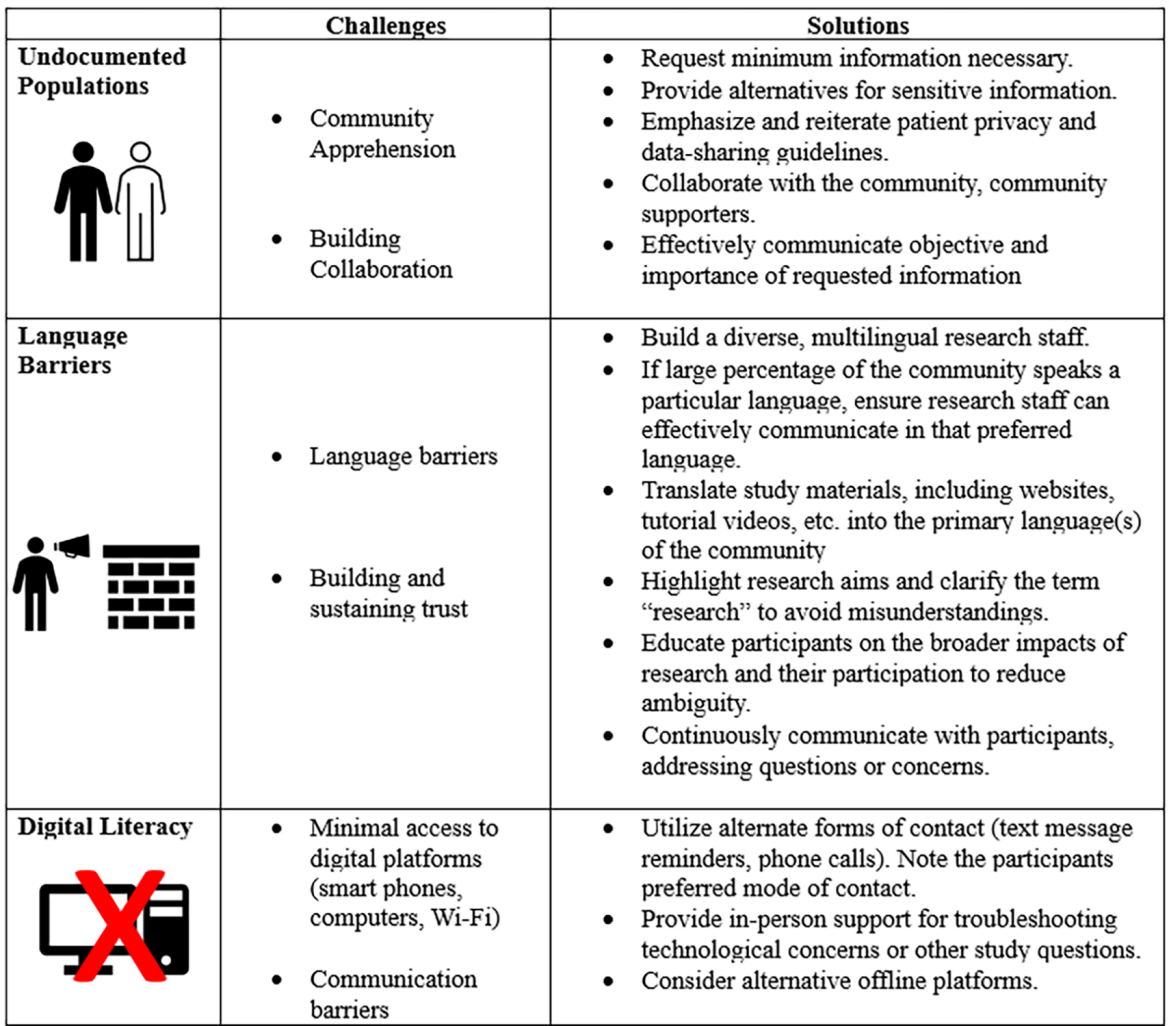
Challenges and solutions for research interventions targeting underserved communities.

Addressing racial and ethnic disparities in social determinants of health, such as FI, requires unique research design approaches (Figure 1). Although there are guidelines and suggestions for performing research on underserved communities (12, 19), implementing them can be challenging. In our experience implementing the FEI intervention, our recruitment efforts were greatly facilitated by our compassionate, empathetic, and culturally sensitive research staff, who were well-equipped to navigate the challenges of engaging vulnerable underserved communities who were largely unfamiliar with biomedical and socio-behavioral research. Obstacles and challenges we faced included how to conduct recruitment in a way that was sensitive to high rates of undocumented status among potential participants, language barriers and poor digital literacy, as well as retaining participants throughout the duration of the study. Here, we provide some specific recommendations for fellow researchers, based on our own experiences engaging local underserved communities in research.

### Supporting undocumented populations

For successful enrollment, consider requesting minimal background information and understand the potential intimidation posed by an institution or organization on these communities. Research may seem intrusive or foreign to communities who would otherwise not partake or engage with it, making potentially apprehensive and anxious undocumented individuals worried about what may happen if they release personal information.

Requesting the minimum necessary information to protect health privacy requires a comprehensive understanding of the community to analyze what areas of research processes may be intimidating. For instance, request only name, email, phone number, and a secondary phone number. However, ensure to obtain what is necessary for your study. For example, our FEI intervention required an address to be provided for food shipments to be delivered. Considering the socioeconomically disadvantaged state of the community and high rates of housing insecurity, an accommodating option is to provide an alternative address, such as a relative's home or work address. However, if a study's objective or hypothesis addresses geospatially distributed factors, an option can be to require only zip codes or other less granular geographic identifiers. In addition, emphasizing, reiterating, and effectively explaining patient privacy and data-sharing guidelines, may further alleviate possible concern or unease. Concurrently explaining the importance and relevance of each requested piece of information during the enrollment process—as well as the relevant data security and legal confidentiality safeguards—can further mitigate persistent concerns around releasing personal information. The collaboration of sensitive and diverse research staff can help discover unique and alternative methods for obtaining critical study information, thereby ensuring adherence to study timelines and objectives.

### Addressing language barriers and building trust with the community

In our study, building trust with participants required proficient multilingual research staff to address language barriers, ensure optimal comprehension of study protocols, participant responsibilities, and available study resources. Prompt and effective communications are essential, therefore multilingual staff should be readily available to address any participant concerns or questions throughout the study, which will be discussed further in a later section (20).

If study participants include non-English speakers, it may be prudent to acquire adequate bilingual trained staff to support their needs and study inquiries. For example, our recruitment efforts targeted the California city of East Palo Alto which is 60.6% Hispanic/Latinx with 64.8% of the population having Spanish as their primary language. Having a Spanish-English bilingual study team with members who previously worked closely in this community during the COVID-19 pandemic allowed us to leverage the previously established community and rapport to connect with a larger portion of the East Palo Alto community. However, if this is not available, consider connecting with a community member(s) who is/are familiar with the community. This can range from local doctors who provide care for those members, school boards, etc. For example, a physician who works in the target community with an already-established rapport can help facilitate trust and familiarity amongst the community, increasing amenability during initial communications. Having fluent Spanish speakers who were previously involved in the community allowed us to better engage potential participants and understand their fears. This greatly reduced participant anxiety about joining a research project or even wanting to get more information on the project itself. Many participants may feel apprehensive at the initial recruitment call, but after explaining who the study team are and that the patient/family were referred to by a trusted physician in their community, they may feel more comfortable continuing the phone call. Effective communication is facilitated when all parties are at ease and confident in their interaction which is critical with engaging in sensitive and vulnerable conversations such as inquiring about FI.

During communications with potential participants, it may be helpful to inquire if there is someone else in the household who speaks English more fluently who can assist in the conversation between the two parties as this can often be a comfortable alternative for families. Having a family member or friend support communications during the early consenting process, logistics, and overall troubleshooting throughout the study can assure participants feel comfortable asking questions, state their concerns, overall assure the participants achieve the proper experience during his study. Some of these concerns include learning how to order the correct food items, understanding the quantities which they will receive, or even clearly understanding the risks and benefits of the study. Having a familiar guide to support study communications can also provide reassurance to study staff that study procedures such as ordering food and completing questionnaires are completed successfully and in a timely manner.

However, this may not always be available and thus it may also be helpful to have a standby translator for other languages of lower frequency within the community of choice. For example, hospitals often have access to an on-call translator service. If this is not available, recruiting multilingual volunteers from a nearby university, can assist other participants that speak other languages that are not as common in the given community. Breaking down the language barriers is important to have clear and easy communication with participants throughout the study and can aid in building and sustaining trust.

Often participants are apprehensive to speak with a non-bilingual staff or one with elementary or limited working proficiency in their language and a translator may create a disconnect between the study team and the participant. When participants can ease through a conversation with a staff member who speaks their native language, it can alleviate some initial stress for them. This creates a comfortable space for the participants to reach out in the future with questions or concerns they may have about the study itself which can also potentially increase enrollment and retention rates. With online components, such as the online market, it is essential to translate the website itself to multiple languages to accommodate participants. If your intervention has not yet had the opportunity to translate your website to the primary language in the target community this may lead to many participants calling the research center for technical support very frequently which could have been prevented by ensuring all intervention resources were translated into the primary languages of the target population earlier on.

Sustaining trust throughout the study is important given the community's frequent lack of familiarity with common research procedures, and the various protections afforded to research participants. It is important to realize that the idea of research may be foreign or intrusive to underserved communities who may have inaccurate or limited knowledge about research and science. Explaining the broadness of the term “research” can help address ambiguity. If research is not culturally understood, “research” may be thought to refer to “wet lab” research where specimens and samples would be acquired for analysis, possible manipulation of samples or study products, or clinical trials where they are not sure whether they will actually receive the desired intervention. In the context of our FEI intervention, for example, we encountered each of these misconceptions. Therefore, we found that clarifying early during the consenting process that research encompasses collecting data via participant questionnaires or interviews to understand their experience and determine the efficacy of study procedures. Being clear that research studies *can* include human sample collection, wet lab use…etc. but that it is not required if contextually irrelevant to the hypothesis and study objective, can calm participants’ fears and misgivings. Clear and effective communication in the participants’ preferred language can strengthen and build community trust, further enhancing the benefits and success of the research interventions.

### Facing poor digital literacy

Poor digital literacy among research participants can present major challenges to researchers during this digital era (21). Poor digital literacy refers to poor exposure to digital technology such as computers, smart phones, and internet-use. Incorporating a digital component can expedite study processes and convenience. However, without adequate guidance and education, a potential participant may have difficulty accessing emails, online questionnaires, website links, or navigating online study platforms, such as online markets. For example, many potential participants found our originally implemented online consenting process puzzling, wherein a uniquely tailored website link was sent to each participant, resulting in a loss in communication, despite seemingly strong interest in study participation.

Text message and phone call communications play a huge role in effective, timely and convenient communication with study participants. Text reminders are beneficial to ensuring participants adhere to study timelines and procedures. Performing check-in phone calls is helpful in identifying potential participant issues that would otherwise have gone unnoticed by participants who are unfamiliar with electronic communications, such as checking their email or spam mailbox which may be uncommon for the participants. Phone calls also play a huge role in troubleshooting participant issues such as navigating online study platforms, accessing their email, locating important study weblinks, addressing how to access their internet (if internet was available to them), how to sign, or even how to submit study documents. Some participants may lack a personal email and may opt to utilize email accounts of their children, spouse, other relatives or even create a new account. This situation frequently required additional support from the research team. As a result, the primary objective of your check-in call may shift to verbally guiding a participant through a process that could be routine for others. Additionally, some participants may lack a smartphone to access weblinks or home internet or potentially limited data plans, which may require them to pay to further access study-related online materials. As a result, they may utilize public facilities such as a community library, child's school, work, or church to access computers and internet. This can make the digital study procedures more burdensome for participants. Moreover, the time required to effectively describe the different website displays depending on the device (iPad, iPhone, PC, Mac, tablet), was burdensome for research staff. As a result, we found such digital troubleshooting calls could require 45–60 min of trained coordinator time to successfully implement, with some calls lasting over an hour. To conclude, while the online study procedures are intended to ease study processes and are often universally assumed to reduce participant and staff burden, we found that this was not always the case. Given these considerations, researchers implementing interventions in communities with low digital literacy should consider factoring in additional technology support for your research project. Nonetheless, taking this time initially to provide technical support, may ultimately benefit both the participants and the study team when sending additional links or emails in the future as participants gradually familiarize themselves with accessibility and utilization of their technology—and could build rapport with the study team.

Hosting office hours at an off-site community space such as a library, church or school can also benefit the research study by providing a comfortable and familiar space for participants. Of note, if you are leading a randomized control trial, these office hours can be set up for control and intervention groups specifically, to avoid participant insights to the randomization process thereby potentially skewing your study results. These office hours can address study related concerns, not just limited to technological concerns, or help walk-through study procedures such as how to place an online order. Being present in the community helps participants feel at ease and provides convenience because of reduced commuting in comparison to an office hour held at the research institution. These office hours can be particularly beneficial during the initial enrollment and consenting process by providing the option of a hardcopy consenting process or virtual one. Not to mention, direct personal interactions with the participant serve to enhance trust and strengthen the relationship. If the study has the option of either electronic or physical gift cards as compensation, these office hours may also be a convenient time and location to distribute these materials.

All in all, while online platforms may seem convenient initially, technology may not always be convenient, reliable, or suitable when engaging with underserved communities. Consequently, some participants may forego study resources which they may otherwise depend on.

## Accessibility of different food support models

### The food prescription (Rx) model

**Figure 2:**
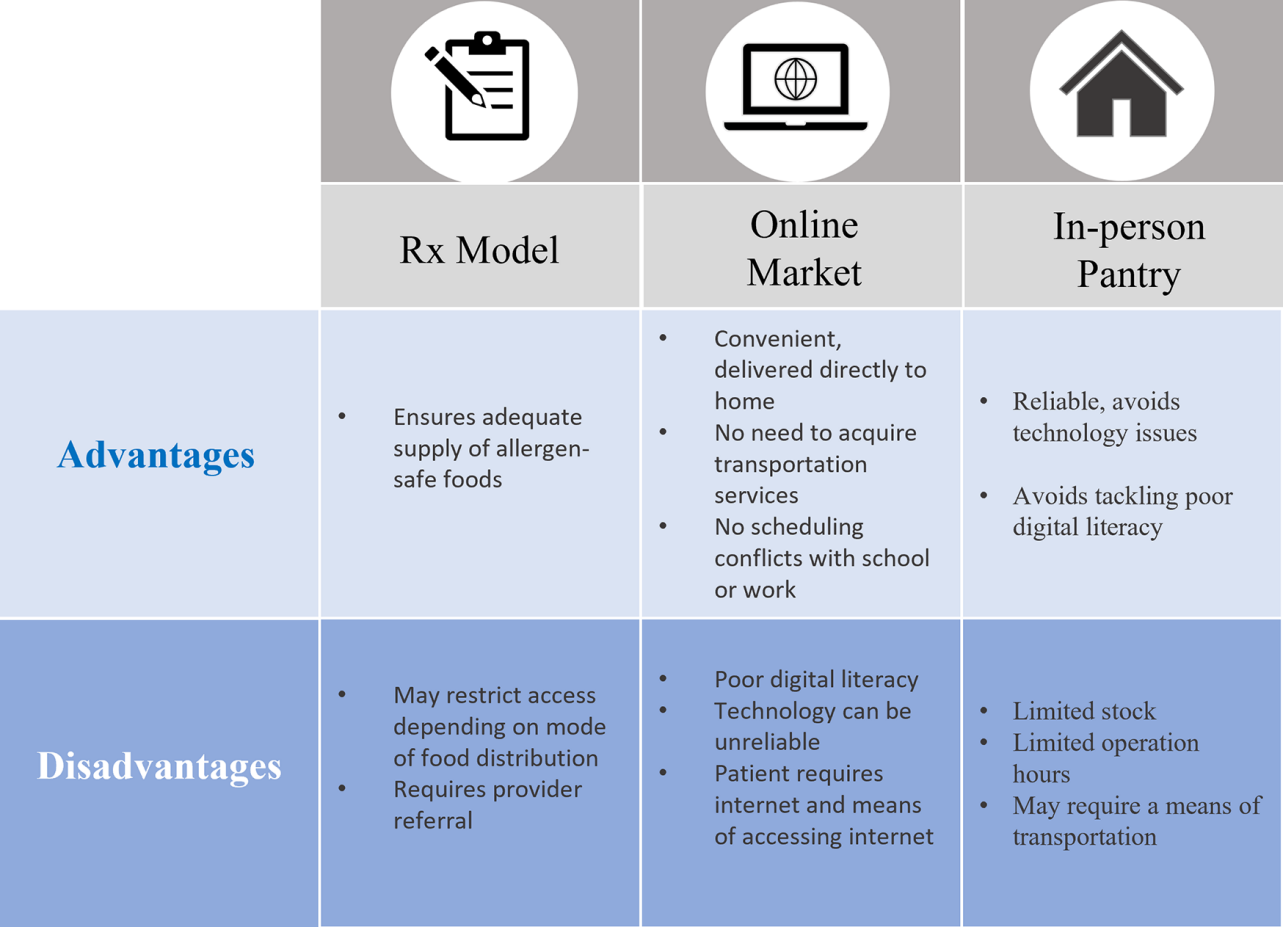
Advantages and disadvantages of intervention models to access to allergen-safe foods for FI families and children.

There are several models for providing food support for participants, each with unique advantages and limitations (Figure 2). While we discuss each model individually here, having different options for participants would increase access and adherence to allergen-safe diets. In the food Rx model, a provider prepares a referral (Rx) to a particular location such as a pantry, grocery store, or even an online market. The participant can take their Rx to their designated location and redeem foods of their choice (22). A food Rx model is helpful to guide participants to a specific food source, but considerations are needed when working with an underserved population.

A previous food Rx study addressing FI successfully decreased the prevalence of FI amongst their participants by 94%. However, FA was not explicitly considered or addressed, despite allowing client-choice when selecting foods (22). Those with FA have greater difficulty identifying safe foods and without allergen-safe options present in the pantries or markets, these options may not accommodate someone with FA. Food-allergic individuals must be diligent in checking food labels to avoid accidental exposure to hidden harmful allergens. For example, some foods may contain nuts for flavoring or other common ingredients such as milk, egg, or wheat. If the goal is to diversify participants’ food intake to achieve a nutritious and balanced diet, an Rx model alone may not be sufficient for all participants.

### Online market platform

An online food market platform (such as Attane-health.com) allows participants to self-select and filter through various allergen-safe food options and have them delivered free of cost. The online platform provides convenience by eliminating commutes to pantries or worry about adhering to food redemption hours. However, the perceived convenience of an online platform may present a significant challenge to communities with poor digital literacy, primarily in underserved neighborhoods.

A key challenge with the online market platform is the heavy reliance on digital literacy and all its associated challenges. To address this, the research team can offer in-person office hours to troubleshoot issues, virtual electronic meetings with screen sharing to guide the participants in real-time, and by preparing instructional videos on how to successfully make a purchase or troubleshoot common issues. Anticipating these challenges is critical in providing solutions for accessibility. Accommodating diverse participants with various helpful resources is prudent when proposing a versatile model system, as one option may not suit the everyone, resulting in the inability to access resources.

### Food pantries

A local pantry may be an optimal supplement to other modes of food redemption by maximizing the options and access to allergen-safe foods for participants. With a physical food pantry in their city, while transportation and scheduling may be a concern, it may be a reliable resource for families as it serves as a back-up or supplemental method. Not to mention, it would add abundance and variety to their food availability at home. For example, if an item was not found on an online food market, they would have the option of browsing their local food pantry.

Shops or pantries with specific “redemption times” may not be accessible to all participants when considering the work lives or financial situations of participants from underserved populations. To provide food-insecure families access to food, a previous study utilized the in-person local food pantries which had designated “redemption times” which are specific pantry hours where the participants could come in for access to allergen-safe foods (22). This study found that their average redemption rates declined from 71.1% to 18.2% over the course of their 6-month program period (22). This lack of attendance was due to the restricted redemption times that did not fit their needs. Often, participants from underserved communities may rely on public transportation or walking as means of transportation which means regularly having to do so may be quite inconvenient or even costly. Therefore, if a family is facing financial strains alongside their FI they may not be in a position to consistently pay for public transportation. Furthermore, these participants may work irregular work schedules, which adds to potential transportation and scheduling concerns with these restricted redemption times. For example, some people may have jobs where they are on “stand-by” waiting for an opportunity such as landscaping or construction side jobs, or some may work very late hours, and while having a redemption time of say 5–7PM twice a week may work for most people working 9AM-5PM, it would not work for someone who is working a night job or even an after-school day care. Another example of what may seem like a convenient redemption time may be the weekend when most people are off from work, but again, this may be optimal work times for a landscaper or construction worker working a side job. Despite this study's decreased redemption rates amongst participants, the intervention decreased FI prevalence by 94%, so it was successful in combating FI despite the unintentional burden it caused for some (22). Finally, it is important to acknowledge potential stigma that may deter a participant from utilizing an in-person pantry. An online format, however, may be beneficial as to combat any negative emotions such as shame and isolation, that may be associated by our families seeking the services of in-person pantries, thereby negatively impacting those already struggling with mental health, especially during the COVID-19 pandemic when food insecure individuals had a 257% higher risk of anxiety and 253% higher risk of depression (23). Offering an alternative online platform is therefore important to consider incorporating to increase utilization by our vulnerable communities. Therefore, a local in-person pantry in conjunction with the option of an online market may be a great way to accommodate a range of FI and FA participants and provide them with a diverse range of allergen-safe foods.

### Post-intervention resources

It is important to provide free sustainable resources for participants post-research intervention to sustain the growing trust, such as partnering with local organizations, or in the case of a food Rx model, a free sustainable food option partnering with local food pantries or FI resources. The FEI study team partnered with a national non-profit organization, Securing Safe Food (SSF), a team dedicated to combating FI in the FA population. SSF stocks U.S food pantries with allergen-safe options so that FI and FA families consistently have a food supply to meet their needs. These families can even sign up for food notifications, informing them when their favorite items are back in stock in their specific pantry. By partnering with a local food pantry, you allow for a sustainable and free option. The FEI research team had a couple patients sign-up for pantry notifications, which notified families when specific items were in stock. If partnering with a similar organization for your study needs is not feasible, consider reaching out to companies and asking for kind donations to ensure your population receives adequate food supply or other resources. Another example for post-intervention resources may be supplying participants with additional resources the research center offers, if any. For example, our FEI participants were welcomed into our clinical research unit for clinical trial prescreening services with our allergist and immunologists if this was of interest to their child's FA. Despite offering a potentially helpful resource, it was difficult for many patients to take time away from work to come the clinical research unit and seek care as it was only open during regular business hours. Of the patients who came into the clinical research unit and were eligible for a clinical trial study, none were able to commit to such a long trial where many full or half days for work and school would be missed. For this reason, it is critical that clinical trials have accommodations and potentially provide safe in-home options or partner with a local community clinic for an alternative location with additional business hours where patients can seek the services provided by a clinical trial. To add, a research team can determine if weekend hours are available at your institution to better accommodate families who cannot spend time away from work. By providing these post-intervention resources as an option, we foster or provide potential continuity with our study participants and larger community. Without sustainable alternatives and guidance, we risk participants disengaging from research altogether, potentially fostering misconceptions and mistrust across future generations, negatively impacting the goal of increasing diversity in research (24). Prior to participants completing the study, one idea for future steps may be to collaborate with a non-profit or community organization to help connect the participants with long-term resources. If partnering with non-profits or local organizations is not feasible, consider referring participants to local organizations and resources. This is especially important when working with vulnerable and sensitive public health concerns such as FI.

If a concrete plan for post-intervention resources and support is not available, it is important to communicate that promptly with the participants. Letting them know that something is in progress helps build camaraderie, and if care is taken to foster strong bonds throughout the study, this message may be well-received. Overall, increasing the diversity of study participants in research helps further our knowledge of their communities, resulting in improved health outcomes.

## Conclusion

In conclusion, incorporating underserved and vulnerable populations into research requires a unique and tailored approach. Successful implementarion of a food-as-medicine intervention in an underserved community is dependent upon a firm understanding of their unique barriers of the community and ensuring adequate solutions are in place to address these barriers. It is essential that a compassionate research team understands the community needs, including supporting undocumented populations, empathy, and proper translation services. Taking steps to ensure participants are comfortable and aware of the study procedures, builds and sustains trust within these communities, potentially resulting in continued engagement with research. Acheiving greater diversity in research cohorts empowers future researchers, physicians and community leaders to gain a deeper understanding of underserved communites and how to optimize their health outcomes.
